# A scalable and unbiased discordance metric with *H*_+_

**DOI:** 10.1093/biostatistics/kxac035

**Published:** 2022-09-05

**Authors:** Nathan Dyjack, Daniel N Baker, Vladimir Braverman, Ben Langmead, Stephanie C Hicks

**Affiliations:** Department of Biostatistics, Johns Hopkins Bloomberg School of Public Health, 615 N Wolfe St, Baltimore, MD 21205, USA; Department of Computer Science, Johns Hopkins University, 3400 N Charles St, Baltimore, MD 21218, USA; Department of Computer Science, Johns Hopkins University, 3400 N Charles St, Baltimore, MD 21218, USA; Department of Computer Science, Johns Hopkins University, 3400 N Charles St, Baltimore, MD 21218, USA; Department of Biostatistics, Johns Hopkins Bloomberg School of Public Health, 615 N Wolfe St, Baltimore, MD 21205, USA

**Keywords:** Clustering, Discordance, Dissimilarity, Single cell

## Abstract

A standard unsupervised analysis is to cluster observations into discrete groups using a dissimilarity measure, such as Euclidean distance. If there does not exist a ground-truth label for each observation necessary for external validity metrics, then internal validity metrics, such as the tightness or separation of the clusters, are often used. However, the interpretation of these internal metrics can be problematic when using different dissimilarity measures as they have different magnitudes and ranges of values that they span. To address this problem, previous work introduced the “scale-agnostic” $G_{+}$ discordance metric; however, this internal metric is slow to calculate for large data. Furthermore, in the setting of unsupervised clustering with $k$ groups, we show that $G_{+}$ varies as a function of the proportion of observations assigned to each of the groups (or clusters), referred to as the group balance, which is an undesirable property. To address this problem, we propose a modification of $G_{+}$, referred to as $H_{+}$, and demonstrate that $H_{+}$ does not vary as a function of group balance using a simulation study and with public single-cell RNA-sequencing data. Finally, we provide scalable approaches to estimate $H_{+}$, which are available in the $\mathtt{fasthplus}$ R package.

## 1. Introduction

Quantifications of discordance such as Gamma (Goodman and Kruskal, 1979) and Tau (Kendall, 1938) have historically been derived to assess fitness from contingency tables. (The terms “discordance” and “disconcordance” have been used interchangeably to describe related metrics for contingency tables (Rohlf, 1974; Goodman and Kruskal, 1979), but here we use “discordance.”)

In this article, we explore the problem of unsupervised clustering (also known as observation partitioning). A typical clustering algorithm seeks to optimally group $n$ observations into $k$ groups (or clusters) using a dissimilarity matrix $D(n \times n)$ (e.g., Euclidean distance) or $d_{ij}$ for each $i$, $j$ observations with $N_{d} = \binom{n}{2}$ unique pairs of distances. If there does not exist a ground-truth label for each observation, internal validity metrics are often used to evaluate the performance of a set of predicted cluster labels $L=[l_{1},\dots,l_{n}:l_{i}=1\dots,k]$ for a fixed $D$. Many internal fitness metrics quantify the tightness or separation of partitions with functions such as within-cluster sums of squares or mean Silhouette scores (Rousseeuw, 1987). However, when comparing multiple dissimilarity measures, the interpretation of these performance metrics can be problematic as different dissimilarity measures have different magnitudes and ranges, leading to different ranges in the tightness of the clusters. 

One solution is to use discordance as an internal validity metric that depends on the ranks of the dissimilarities, rather than on the dissimilarities themselves, thereby making it a “scale-agnostic.” For example, the discordance metric $G_{+}$ (Williams and Clifford, 1971; Rohlf, 1974) uses the following to assess how well a given predicted cluster label $L$ fits a dissimilarity $D$ induced from the same observations (Rohlf, 1974; Desgraupes, 2018) (Note 1 of the Supplementary material available at *Biostatistics* online):

(1.1) \begin{align*}
s = \sum_{i=2}^{n} \sum_{j<i} 1_{[a_{ij}=1]} \sum_{u=2}^{n} \sum_{v<u} 1_{[a_{uv}=0]} 1_{[d_{ij}>d_{uv}]}
\end{align*}

given fixed $L$, an adjacency matrix $A(n \times n)$ is defined using the predicted cluster label $l_i$, $l_j$ for the $i$, $j$ observations, where $a_{ij} = 1$ if $l_{i}=l_{j}$ or $a_{ij} = 0$ otherwise. We can define the set of within-cluster distances as $D_{W}=\{d_{ij}:a_{ij}=1;i=2,\dots,n,j<i\}$ and between-cluster distances as $D_{B}=\{d_{uv}:a_{uv}=0;u=2,\dots,n,v<u\}$ with the total number of distances in each set as $\lvert D_{W}\rvert$ and $\lvert D_{B}\rvert$, respectively. As we know that each upper triangular entry of $A$ is binary (every distance is either between- or within-cluster), then $\lvert D_{W}\rvert+\lvert D_{B}\rvert=N_{d}$. Here, we define $\alpha$ as the proportion of total distances $N_{d}$ that are within-cluster distances, or $\alpha = \frac{\lvert D_{W}\rvert}{N_{d}}$.

**Fig. 1:**
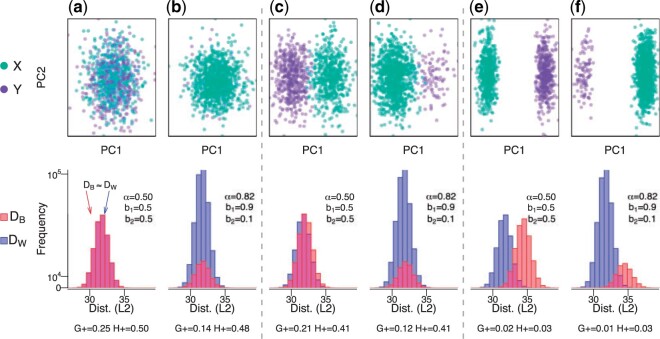
The $G_{+}$ discordance metric varies as function of $\alpha$ (proportion of within-cluster distances), which is a function of the group balance. We randomly sampled $n$ = 1000 observations with 500 features from a mixture distribution $b * X + (1-b) * Y$ with $b$ being the probability of an observation coming from $X \sim N(\mu_x, \sigma^2)$ and $1-b$ coming from $Y \sim N(\mu_y, \sigma^2)$ with (a,b) no mean difference ($\mu_x - \mu_y = 0$) (or a “null” setting), (c,d) a small mean difference ($\mu_x - \mu_y = 0.2$), and (e,f) a large mean difference ($\mu_x - \mu_y = 0.6$). We simulate data with (a,c,e) balanced groups ($b$ = 0.5) and (b,d,f) imbalanced groups ($b$ = 0.9). For each simulation, the top row contains observations belonging to a group ($X$ and $Y$) along the first two principal components (PCs) and the bottom row contains histograms of the within- ($D_{W}$) and between- ($D_{B}$) cluster distances (Euclidean) for the balanced and imbalanced groups. Refer to Figure S1 of the Supplementary material available at *Biostatistics* online for an illustration of (and Section 2.4.1 for the explicit relationship between) the proportion of within-cluster distances ($\alpha$) and the group balance ($b$). For each simulation, the bottom row includes $\alpha$ and the two discordance metrics $G_{+}$ and $H_{+}$. Generally, values close to zero represent more concordance, while a larger values represent more discordance.

In the following sections, we first consider properties of $G_{+}$ and show how $G_{+}$ is a function of $\alpha$ (Section 2), which has an explicit relationship with what we refer to as $b$ (the group balance, Section 2.4.1), where $b_k$ is the proportion of observations assigned to each of $k$ groups (or clusters) and $\sum_k b_k = 1$. We illustrate how this is an undesirable property for $G_{+}$ to vary as a function of $\alpha$, thereby also the vector $b$ and $k$. For example, when simulating “null” data (random Gaussian data with no mean difference between $k=2$ groups), the expected mean (and the interpretation itself) of the $G_{+}$ discordance metric varies depending on $b$ (e.g., if the groups are balanced or $b = (0.5, 0.5)$, then $G_{+} = 0.25$, but if the groups are imbalanced, such as $b = (0.9, 0.1)$, then $G_{+} = 0.14$ using simulated data) (Figure 1). In addition, we demonstrate that $G_{+}$ is slow to calculate for large data (due to the pairwise comparisons of dissimilarities in (1.1)). To ameliorate these challenges, we propose a modification to $G_{+}$, referred to as $H_{+}$ (Section 3) and demonstrate that $H_{+}$ does not vary as a function of group balance using a simulation study and with public single-cell RNA-sequencing (scRNA-seq) data (Section 4). Finally, we provide scalable approaches to estimate $H_{+}$, which are available in the $\mathtt{fasthplus}$ R package. 

## 2. The $G_+$ discordance metric

The discordance metric $G_{+}$ (Williams and Clifford, 1971; Rohlf, 1974) scales $s$ (1.1) by $\binom{N_d}{2}$, the number of ways to compare each unique distance to every other.

(2.2) \begin{equation*}
G_{+}=\frac{s}{N_{d}(N_{d}-1)/2}.
\end{equation*}

Generally, $G_{+}$ close to zero represents high concordance, while a larger $G_{+}$ is more discordant. In this way, $G_{+}$ can be used to quantify the cluster fitness for a given $D$ and $L$ (that is, a designation of each pairwise dissimilarity as within- or between-cluster), where a small $G_{+}$ value would be interpreted as good performance with tight, separate clusters. 

### 2.1. Applications of $G_+$

As noted above, if $D$ is fixed, smaller values of $G_{+}$ among many sets of labels $L_{1}, L_{2} \dots$ indicate increased cluster fitness (or the generated labels with smaller $G_{+}$ have more accurately described the dissimilarity structure of the data) (Rand, 1971; Williams and Clifford, 1971). If we instead fix $L$, we can also use $G_{+}$ to assess the fitness of multiple dissimilarity matrices $D_{1}, D_{2}\dots$ (Rohlf, 1974). 

Because $G_{+}$ depends on the relative rankings of pairwise distances, this transformation enables a “scale-agnostic” approach to compare dissimilarity measures through the structure they impose on the data, rather than by the exact values of the distances themselves. This allows distances to be compared on varying scales without imposing bias from the expected magnitude of distances. 

### 2.2. Properties of $s$

Consider $s$ (1.1) with an adjacency matrix $A(n \times n)$ and dissimilarity matrix $D(n \times n)$, with induced within-cluster distances $D_{W}$ and between-cluster distances $D_{B}$ where $\lvert D_{W}\rvert+\lvert D_{B}\rvert=N_{d}$. We can define $\alpha \in (0,1)$ as the proportion of total distances $N_{d}$ that are within-cluster distances. In this way, $\lvert D_{W}\rvert=\alpha N_{d}$, and similarly, $\lvert D_{B}\rvert=(1-\alpha)N_{d}$. Then, conditional on $N_{d}$ and $\alpha$, the $E[s]$ is (Note 1 of the Supplementary material available at *Biostatistics* online):

(2.3) \begin{equation*}
E[s] = \alpha(1-\alpha)N_{d}^{2} P(d_{ij}>d_{uv}),
\end{equation*}

where $P(d_{ij}>d_{uv})$ is the probability that a within-cluster distance $d_{ij}\in D_{W}$ is greater than a between-cluster distance $d_{uv}\in D_{B}$. This is the quantity we are interested in estimating, but there is a scaling factor $\alpha(1-\alpha)N_{d}^{2}$ that depends on both $\alpha$ and $N_d$. Next, we consider properties of first $P(d_{ij} > d_{uv})$ and then $G_{+}$.

### 2.3. Properties of $P(d_{ij} > d_{uv})$

If we know the expected mean and variance for $d_{ij}\in D_{W}$ and $d_{uv}\in D_{B}$, we can estimate $P(d_{ij}>d_{uv})$. In the simple case where $E[D_{W}]=E[D_{B}]$, we can consider $X=D_{W}-D_{B}$, then $E[X]=0$ and a standardization of $X$ demonstrates (assuming [co]variances exist) that $P(D_{W} - D_{B}>0) = \frac{1}{2}$. As we might expect, there is a $50\%$ chance that $d_{ij}>d_{uv}$ when $E[D_{W}]=E[D_{B}]$. 

### 2.4. Properties of $G_+$

Using (2.2) and (2.3), the expected value of $G_{+}$ is (Note 1 of the Supplementary material available at *Biostatistics* online):

\begin{equation*}
E[G_{+}] = \frac{N_{d}}{N_{d}-1}2\alpha(1-\alpha)P(d_{ij}>d_{uv})
\end{equation*}

As $\frac{N_{d}}{N_{d}-1} \to 1$ for large enough $N_{d}$, then we see that $E[G_{+}]$ is a function of $2\alpha(1-\alpha)$. Next, we derive the relationship between $\alpha$ and $b$ (group balance) (Section 2.4.1). Then, we provide an illustration of how $G_{+}$ varying as function of $\alpha$ and $b$ is an undesirable property (Section 2.4.2). 

#### 2.4.1. Relationship between $\alpha$ and $b$

Herein, we derive the relationship between $\alpha$ (the proportion of total distances $N_d$ that are within-cluster distances) and the group balance $b$ (the proportion of observations assigned to each of the $k$ groups). For an arbitrary label $L$ (a vector of length $n$, $L_{i}\in1,\dots,k$) where $L_{i}=j$ indicates that the $i{\rm th}$ observation is assigned membership to the $j{\rm th}$ cluster group, we can define the portion of observations in group $j$ using $b_j$ defined as

\begin{equation*}
b_{j}=\frac{1}{n}\sum_{i = 1}^{n} 1_{[L_{i}=j].}
\end{equation*}

By definition, we know $\sum_{j=1}^{k} b_{j} = 1$. Each of the $k$ clusters will contribute to the quantity $\alpha$, which is a fraction of the $N_d = n(n-1)/2$ unique pairs of distances. Now, for the $j{\rm th}$ cluster, this contribution ($c_j$) is the upper triangular elements of a matrix block with size $n\cdot b_{j}$

\begin{equation*}
c_{j}=\frac{nb_{j}(nb_{j}-1)}{2}.
\end{equation*}

Finally, we can express $\alpha$ as a sum over each of $k$ contributions $c_{j}$ for $j=1,\dots,k$ for the explicit relationship between $\alpha$ and the $b_j$s (and consequently $k$)

(2.4) \begin{equation*}
\alpha=\frac{2}{n(n-1)}\cdot\sum_{j=1}^{k}c_{j}=\frac{2}{n(n-1)}\cdot\sum_{j=1}^{k}\frac{nb_{j}(nb_{j}-1)}{2}=\frac{1}{n-1}\sum_{j=1}^{k}b_{j}(nb_{j}-1).
\end{equation*}

#### 2.4.2. $G_{+}$ as a function of $\alpha$ and $b$ is an undesirable property

Because $G_{+}$ is a function of $\alpha$ and thereby the group balance $b$ (and consequently $k$), the interpretation of what we expect $G_{+}$ to mean, for example, in a null setting without any true difference between groups, changes across data sets with different group balances $b$. 

For example, assume we randomly sampled $n$ = 1000 observations with 500 features from a mixture distribution $b*N(\mu_x, \sigma^2) + (1-b)* N(\mu_y, \sigma^2)$ with no mean difference ($\mu_x - \mu_y = 0$) and balanced classes ($b$ = 0.5 and $\alpha$ = 0.5) then, we know $P(d_{ij}>d_{uv}) = \frac{1}{2}$ and $E[G_{+}] = 2 \frac{1}{2} (1-\frac{1}{2}) \frac{1}{2} = \frac{1}{4}$. This can be thought of as a “null” simulation where we expect no difference in class character or balance, yet $G_{+}$ will (perhaps unintuitively) equal $\frac{1}{4}$. However, if there is an imbalance in class sizes ($b$ = 0.9 and $\alpha$ = 0.82) then $E[G_{+}] \approx 2 \cdot 0.82 \cdot (1-0.82) \cdot \frac{1}{2} \approx 0.14$ (Figure 1). An illustration of the relationship between $\alpha$ and $b$ for this example can be seen in Figure S1A,B of the Supplementary material available at *Biostatistics* online, which shifts the majority of the distances to within-cluster distances simply due to the imbalance of the classes. 

However, if we consider the same scenario as above, but if we change $k$ from $k=2$ to $k=10$, we see that because there are a larger number of groups, this changes $\alpha$ (the portion of within-cluster distances) for both the balanced (Figure S1C of the Supplementary material available at *Biostatistics* online) and imbalanced simulations (Figure S1D of the Supplementary material available at *Biostatistics* online). 

## 3. The proposed method

### 3.1. An unbiased discordance metric with $H_+$

To ameliorate this effect, we propose $H_{+}$, which replaces the scaling factor $N_{d}(N_{d}-1)/2$ in the denominator in $G_{+}$ with $\lvert D_{W}\rvert \lvert D_{B}\rvert = \alpha(1-\alpha)N_{d}^{2}$:

(3.5) \begin{equation*}
H_{+}=\frac{s}{\lvert D_{W}\rvert \lvert D_{B}\rvert}.
\end{equation*}

In other words, instead of scaling $s$ by the total number of ways to compare every distance to every other distance, we divide by the number of ways to compare within-cluster distances to between-cluster distances. Hence, $E[H_{+}]$ is not a function of $\alpha$:

\begin{equation*}
E[H_{+}]=\frac{\alpha(1-\alpha)N_{d}^{2}P(d_{ij}>d_{uv})}{\alpha(1-\alpha)N_{d}^{2}}=P(d_{ij}>d_{uv}).
\end{equation*}

**Fig. 2:**
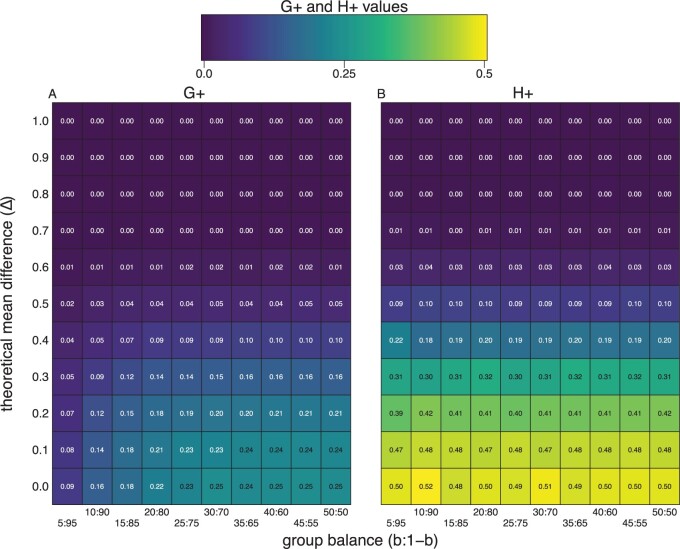
The $H_{+}$ discordance metric does not change as a function of class balance. We randomly sampled $n$ = 1000 observations with 500 features from a mixture distribution $b * X + (1-b) * Y$ with $b$ being the probability of an observation coming from $X \sim N(\mu_x, \sigma^2)$ and $1-b$ coming from $Y \sim N(\mu_y, \sigma^2)$ with a true mean difference ($\Delta = \mu_x - \mu_y$) ($y$-axis). Along the $x$-axis we change group (or class) balance from balanced (e.g., $b$ = 0.50) and to imbalanced (e.g., $b$ = 0.05) groups. The plots are heatmaps of true $G_{+}$ (left) and $H_{+}$ (right) discordance metrics, which shows $H_{+}$ does not change as a function of class balance ($x$-axis), only as a function of the true effect size ($y$-axis).

In fact, we can empirically verify that while $G_{+}$ varies as a function of $\alpha$ (and $b$) (Figure 2a), $H_{+}$ does not (Figure 2b), regardless of difference in expectation between the groups. 

### 3.2. Generalizing properties of $P(d_{ij} > d_{uv})$

More generally, consider the function $1_{[d_{ij}>d_{uv}]}$. For some constant $c$, we can decompose this event as a joint event $1_{[d_{ij}>c]} \cap 1_{[d_{uv}\leq c]}$ or $1_{[d_{ij}>c]} \cap (1-1_{[d_{uv}>c]})$ (Jardine and Sibson, 1968; Rohlf, 1974). Therefore, as $E[H_{+}]=P(d_{ij}>d_{uv})$, we can decompose $E[H_{+}]$ into two quantities: $E[H_{+}]=\gamma_{W}\gamma_{B}$ where $\gamma_{W} = P(d_{ij}>c)$ and $\gamma_{B} = (1-P(d_{uv}>c))$. In other words, $H_{+}$ empirically states a $100\times\gamma_{W}\%$ of $d_{ij}\in D_{W}$ is strictly greater than $100\times\gamma_{B}\%$ of $d_{uv}\in D_{B}$. This implies $H_{+}$ is not uniquely determined. For example, if $H_{+}=0.4$, we could have $\gamma_{W}=1.00,\gamma_{B}=0.4$ or $\gamma_{W}=0.80,\gamma_{B}=0.50$. It should be noted that one can construct examples where two distinct pairs of $\gamma_{W},\gamma_{B}$ will have the same product, but do not imply each other. 

### 3.3. Two algorithms to estimate $H_+$ and $\gamma_{W}$, $\gamma_{B}$

**Fig. 3:**
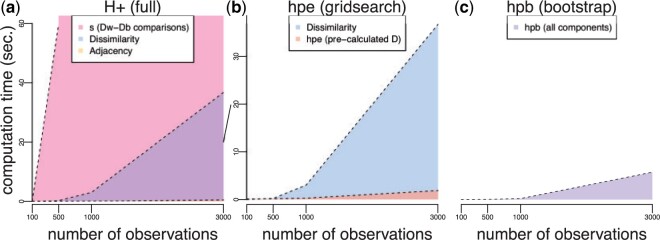
Computation times (seconds) for exact and approximate $H_{+}$ calculations as a function of increasing number of observations $n$. Computational time ($y$-axis) as a function of observations ($x$-axis) to calculate the individual components of (a) exact $H_{+}$ including (i) the dissimilarity matrix $D(n \times n)$ (purple) scaling $O(n^2)$, (ii) the adjacency matrix (orange), and (iii) the most expensive operation $s$ (pink) scaling $O(n^4)$. Note, $s$ is only shown for $n$ = 100 and 500 observations, but the trend is shaded in for the other observations; (b,c) have different *y*-axes than (a). The diagonal line between (a) and (b) connects the 20-s ticks of these two axes. (b) Approximate $H_{+}$ estimation (HPE) using the grid search procedure including (i) the dissimilarity matrix $D(n \times n)$ (blue) scaling $O(n^2)$ and (ii) the HPE algorithm to estimate $s$ (orange) scaling $O(p)$; (c) approximate $H_{+}$ estimation using the bootstrap procedure (HPB) (purple), which scales similarly to HPE without the computational expense required for calculating $D$. Note (b) and (c) have a different $y$-axis scale than (a) for an zoomed in visualization of time.

One problem with the $H_{+}$ (and $G_{+}$) discordance metric (3.5) is that it requires the calculation of both (i) the dissimilarity matrix $D(n\times n)$ which scales $O(n^2)$ and (ii) $s$ (1.1) which scales with the number of ways to compare within-cluster distances to between-cluster distances (or $O(n^4)$ comparisons). For example, with data sets of sizes $n$ = 100 and 500, it takes 0.01 and 0.22 s, respectively, to calculate $D(n\times n)$ and it takes 0.08 and 59.68 s, respectively, to calculate $s$ (Figure 3a, Table S1 of the Supplementary material available at *Biostatistics* online). For data sets with more than $n$ = 500 observations, this quickly becomes computationally infeasible. 

To address this, we propose two algorithms to estimate $H_{+}$, both referred to as an “h-plus estimator” or (HPE): (i) a brute force approach inspired by the Top-Scoring Pair (Leek, 2009; Magis and Price, 2012) algorithms, which use relative ranks to classify observations with $O(p^2)$ comparisons and (ii) a grid search approach with $O(p)$ comparisons, where $p$ refers to percentiles of the data (rather than the $n$ observations themselves). Typically, $p$ is chosen such that $p < n$, leading to significant improvements in the computational speed to calculate $H_{+}$. Specifically, both algorithms estimate $H_{+}$ (referred to as $H_{e}$ or HPE) assuming $D(n \times n)$ has been precalculated and provide faster ways to approximate $s$ (Figure 3b). Both algorithms are implemented in the hpe() function in the fasthplus R package. 

Finally, in a later section (Section 3.5), we introduce a third algorithm based on bootstrap sampling to avoid calculating the full dissimilarity matrix $D$, thereby leading to further improvements in computational speed to estimate $H_{+}$ (referred to as $H_{b}$ or HPB) (Figure 3c). The bootstrap algorithm is implemented in the hpb() function in the fasthplus R package. 

#### 3.3.1. Intuition behind HPE algorithms

The estimator $H_{e}$ (or HPE) assumes $D(n \times n)$ has been precalculated and then provides faster ways to approximate $s$ (the pairwise comparisons of $D_W$ and $D_B$). Specifically, we let the two sets $D_{W}$ and $D_{B}$ represent the ordered (ascending) dissimilarities $d_{ij}\in D_{W}$ and $d_{uv}\in D_{B}$, respectively. Then, we bin the sets $D_{W}$ and $D_{B}$ into $p+1$ percentiles where $q(D_{W})_{i}$ and $q(D_{W})_{j}$ are the percentiles for $i,j=0,\dots,p$. Note, $q(D_{W})_{0}=\min(D_{W})$ and $q(D_{W})_{p}=\max(D_{W})$. In both algorithms below, we check if $q(D_{W})_{0}>q(D_{B})_{p}$, then $H_{e}=1$, and similarly, if $q(D_{W})_{p}<q(D_{B})_{0}$ then $H_{e}=0$. 

**Fig. 4:**
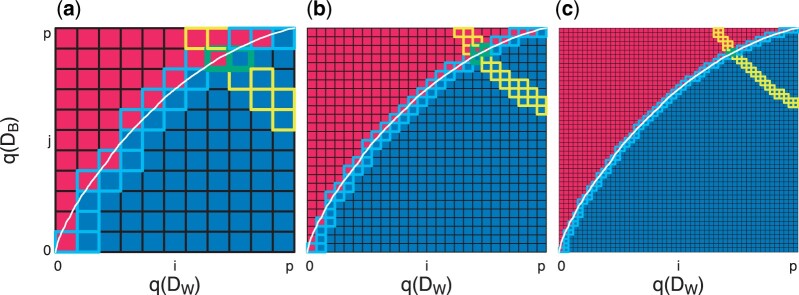
Graphical representation of two HPE algorithms to estimate $H_{+}$. We simulate observations from two Gaussian distributions, namely $A \sim N_{10\,000}(0.3,1)$ and $B\sim N_{10\,000}(-0.3,1)$ and calculate the quantiles $q(D_{W})$ and $q(D_{B})$ for each of the sets with (a) $p+1 = 11$, (b) $p+1 = 26$, and (c) $p+1 = 51$. The white curve represents the percent of elements in $D_{W}$ that are strictly less than each element in $D_{B}$. The goal is to estimate the true $H_{+} (\approx 0.66)$ (area under the white curve) using one of two HPE algorithms. The brute force approach (HPE algorithm 1) uses Riemann integration to approximate the white curve by summing the area of the blue squares below the curve. The grid search approach (HPE algorithm 2) starts at the minimum of $q(D_{W})$ and $q(D_{B})$ and moves along the red–blue border to approximate the white curve (path followed represents the squares with the light blue borders). The HPE contour $H_{e}$ (or estimate of $H_{+}$) $\pm\frac{1}{p-1}$ is given by yellow-bordered squares. In other words, every pair $\gamma_{W},\gamma_{B}$ such that $\gamma_{W}\cdot\gamma_{B} \in H_{e}\pm\frac{1}{p-1}$, the interval guaranteed to contain $H_{+}$. The intersection of this yellow contour ($H_{e}\pm\frac{1}{p-1}$) and blue contour (grids visited by HPE algorithm 2) are the green-bordered squares, which represents the numerical estimate for $\gamma_{W}$ and $\gamma_{B}$.

Next, we provide a graphical intuition for the two HPE algorithms by performing a simulation study. First, we simulate observations from two Gaussian distributions, namely $D_{W} \sim N_{10\,000}(0.3,1)$ and $D_{B} \sim N_{10\,000}(-0.3,1)$ and calculate the quantiles $q(D_{W})$ and $q(D_{B})$ for each of the sets with $p+1 = 11$ (Figure 4a), $p+1 = 26$ (Figure 4b), and $p+1 = 51$ (Figure 4c). The calculation of these quantiles seeks to approximate the true ordered inequality information for each $d_{ij}\in D_{W}$ and $d_{uv}\in D_{B}$. That is, if $D_{W},D_{B}$ were both given in ascending order, the white line in Figure 4 shows the percent of $d_{uv}\in D_{B}$ that is strictly less than each $d_{ij}\in D_{W}$. The true $H_{+} (\approx 0.66)$ is then given by the area under the white curve (the true rank orderings for each pair). Our goal is to use the following two algorithms to estimate the true $H_{+}$ (fraction of blue area in the grid). 

#### 3.3.2. HPE algorithm 1: $H_+$ (brute force)

Algorithm 1 numerically approximates $H_{+}$ with Riemann integration. Specifically, using a double $\mathtt{for}$ loop with $(p+1)^{2}$ comparisons, this brute force approach sums the area of the squares that are blue in Figure 4, resulting in an algorithm on the order of $O(p^2)$. The path taken by our implementation of this algorithm is given by the squares with light blue borders, and the contour corresponding to the true $H_{+}\approx0.66$ is (approximately) represented by the squares with yellow outlines (Figure 4).

Algorithm 1
$H_{+}$ (brute force)

1. $s_{e} = 0$
2. **for**$i=0,\dots,p$**do**
3.       **for**$j=0,\dots,p$**do**
4.             $s_{e} += 1(q(D_{W})_{i}>q(D_{B})_{j})$
5.       **end for**
6. **end for**
7. $H_{e}=s_{e}/p^{2}$

#### 3.3.3 HPE algorithm 2: $H_+$ (grid search)

An alternative and faster approach (on the order of $O(p)$ comparisons) is to sketch the surface (blue–red border) that defines $H_{+}$. By starting at the minimum of $q(D_{W})$ and $q(D_{B})$, Algorithm 2 moves along the blue–red border that defines $H_{+}$ using grid search to determine whether to increase $q(D_{W})_{i}$ or $q(D_{B})_{j}$ with each iteration.

Algorithm 2
$H_{+}$ (grid search)

 1. $s_{e} = 0$
 2. $s_{e} = 0$
 3. $i=j=0$
 4. $v=[0,0,\dots,0]$
 5. **while**$i=<p$ and $j=<p$**do**
 6.      $s_{i}=q(D_{W})_{i}>q(D_{B})_{j}$
 7.      **if**$s_i$**then**
 8.        $j++$
 9.      **else**
10.        $z_{i} = j/p^{2}$
11.        $i++$
12.      **end if**
13. **end while**
14. $H_{e}=\sum_{i=0}^{p}z_{i}$

### 3.4. Convergence of HPE algorithms 1 and 2

Next, we provide a numerical bound for the accuracy of $H_{e}$ for both the brute force and grid search approaches. For each $q(D_{W})_{i}$, $i=0,\dots,p$, HPE algorithm 2 (and intrinsically algorithm 1) ascertains one of the following:

(3.6) \begin{equation*}
\begin{array}{ccc}
(1) & q(D_{W})_{i}<q(D_{B})_{0}\\
(2) & \exists j:q(D_{B})_{j}<q(D_{W})_{i}\leq q(D_{B})_{j+1} & j\in0,\dots,p-1\\
(3) & q(D_{W})_{i}>q(D_{B})_{p}
\end{array}
\end{equation*}

In (1), we have confirmed that $\frac{i}{p}\times100\%$ of $D_{W}$ are less than $\frac{0}{p}\times100\%$ of $D_{B}$ and the $i^{th}$ addition to the numerical integral will be zero, that is, $z_{i}=0$ in HPE algorithm 2. In (3), we see that $\frac{i}{p}\times100\%$ of $D_{W}$ are greater than $\frac{p}{p}\times100\%$ of $D_{B}$ and $z_{i}=\frac{1}{p}$ in HPE algorithm 2. In (2), we know that $\frac{i}{p}\times100\%$ of $D_{W}$ are bigger than $\frac{j}{p}\times100\%$ of $D_{B}$, but not greater than $\frac{j+1}{p}\times100\%$ of $D_{B}$, and $z_{i}=\frac{j}{p^{2}}$ in HPE algorithm 2. Recall that $H_{e}$ is estimated as the sum over each $z_{i}$ where $\frac{j}{p^{2}}\leq z_{i}<\frac{j+1}{p^{2}}$. We denote $y_{i}$ as the true value of this sum for column $i$, that is, for some $\beta$, $y_{i}=\frac{\beta}{p^{2}}$ where $\frac{\beta}{p}\times100\%$ of $D_{B}$ are less than or equal to $q(D_{W})_{i}$ and $\frac{j}{p^{2}}<y_{i}\leq\frac{j+1}{p^{2}}$. Thus, for (2), we have the condition $0\leq|z_{i}-y_{i}|<\frac{j+1}{p^{2}}-\frac{j}{p^{2}}=\frac{1}{p^{2}}$, in other words, the addition to $H_{e}$ from the $i{\rm th}$ column will differs from the true value ($H_{+})$ by at most $\frac{1}{p^{2}}$. Thus, for all $p$:

(3.7) \begin{equation*}
\lvert H_{+}-H_{e}\rvert \leq\sum_{i=0}^{p}|z_{i}-y_{i}|\leq(p+1)\frac{1}{p^{2}}<\frac{1}{p}.
\end{equation*}

That is, by taking $p+1$ percentiles of $D_{W}$ and $D_{B}$, our estimate for HPE algorithm 2 will be within $\frac{1}{p}$ of $H_{+}$. This follows when one considers HPE algorithms 1 and 2 are approximations of the paired true rank comparisons (white curve in Figure 4) using Riemann integration with increasing accuracy as a function of $p$. An additional argument for the convergence of these algorithms is presented in Note 2 of the Supplementary material available at *Biostatistics* online. 

#### 3.4.1. Estimating $\gamma_{W}$ and $\gamma_{B}$

To estimate $\gamma_{W}$ and $\gamma_{B}$, we use the intersection of the yellow contour ($H_{e}\pm\frac{1}{p-1}$) and blue contour (path visited by HPE algorithm 2), which are the green-bordered squares in Figure 4. As our approach guarantees that $H_{e} - \frac{1}{p-1} \leq H_{+} \leq H_{e} + \frac{1}{p-1}$, we can identify every pair $\gamma_{W},\gamma_{B}: \gamma_{W}\cdot \gamma_{B} \in H_{e} \pm \frac{1}{p-1}$ as potential values of $\gamma_{W},\gamma_{B}: \gamma_{W}\cdot \gamma_{B} \approx H_{+}$. Our algorithm also identifies the values of $\gamma_{W},\gamma_{B}$ that are true for the observed data (all areas below the white line in Figure 4) or those which have been verified as false (all area above the white line in Figure 4). Our estimate for $\gamma_{W},\gamma_{B}$ is then the intersection of $\gamma_{W},\gamma_{B}$ that are empirically verified in HPE algorithm 2 such that $100\times\gamma_{W}\%$ of $d_{ij}\in D_{W}$ is strictly greater than $100\times\gamma_{B}\%$ of $d_{uv}\in D_{B}$ (blue squares in Figure 4) and $\gamma_{W},\gamma_{B}$ which satisfy $\gamma_{W}\cdot \gamma_{B} \approx H_{+}$ (yellow squares in Figure 4). 

### 3.5. Bootstrap algorithm to estimate $H_+$

As noted in Section 3.3, while the computational speed of the HPE algorithms for identifying ways to approximate $s$ is significantly faster than calculating the full $H_{+}$ (Figures 3(a) and (b)), both of these algorithms assume the dissimilarity matrix $D(n\times n)$ has been precomputed and that an adjacency matrix $A(n \times n)$ must be calculated. Unfortunately, the $O(n^{2})$ computational requirements for full pairwise dissimilarity calculation to quickly becomes infeasible (Figure 3, Table S1 of the Supplementary material available at *Biostatistics* online). 

To address the limitation of computing and storing all pairwise dissimilarities, we implemented a bootstrap approximation of $H_{+}$ (HPB or $H_{b}$) that samples with replacement from the original $n$ observations $r$ times (bootstraps) with a per-bootstrap sample size $t$. We sample proportionally according to the vector $b$ as described in Section 2.4.1, that is, each of the $k$ clusters is randomly sampled $t_{j} \approx b_{j}\times t$ times (where $\sum_{j=1}^{k}b_{j}=1$) such that $\sum_{j=1}^{k}t_{j}\approx t$. For each of $r$ iterations, the $t$ sampled observations are used to generate dissimilarity and adjacency matrices which are then used to calculate a point estimate of $H_{+}$. The mean over these $r$ bootstraps is $H_{b}$, the bootstrap $H_{+}$ estimate. The bootstrap approach scales substantially better than full dissimilarity calculation (Figure 3c). In our simulations, bootstrap parameters $r=0.05\times n$, $t=100$ yield $H_{+}$ estimates within $0.01$ of that given by HPB with $p=10\, 001$ ($1e-04$ accuracy) with economical performance improvements. For example, we saw a reduction in computation time from $38.57$ s with HPE to $5.74$ s with HPB at 3000 observations) (Figure S2 and Table S1 of the Supplementary material available at *Biostatistics* online). 

## 4. Application of $H_+$ to the analysis of single-cell RNA-sequencing data

In this section, we demonstrate the use of $H_{+}$ as an internal validity metric in the application of scRNA-seq data with predicted cluster labels. Also, we compare $H_{+}$ to other widely used validity measures, including both (i) external (i.e., comparing predicted labels to ground-truth clustering known *a priori*) and (ii) internal (derived from the data itself) measures (Halkidi *and others,* 2001; Theodoridis and Koutroumbas, 2008). 

### 4.1. Motivation

Consider a scRNA-seq data set with $n$ observations (or cells) each with $G$ features (or genes). We introduced and formulated $H_{+}$ an internal validity metric to assess the fitness of a single dissimilarity measure $D$ and label $L$. Here, we introduce two scenarios where the goal is to compare the performance of either (i) two label sets $L_{m}$, $L_{m-1}$ and a fixed dissimilarity $D$ or (ii) two dissimilarity measures $D_{m}$, $D_{m-1}$ with a fixed label $L$. In the first scenario, $m$ and $m-1$ could represent two iterations in a single clustering algorithm or they could be labels from two separate clustering algorithms. As $E[H_{+,m}]=P(d_{ij}>d_{uv})_{m}$ (and similarly with $m-1$), the condition $H_{+,m}<H_{+,m-1}$ can be rewritten as follows

(4.8) \begin{equation*}
P(d_{ij}>d_{uv})_{m} < P(d_{ij}>d_{uv})_{m-1}.
\end{equation*}

As $N_{d}$ is fixed in the following subsections, we offer interpretations of the condition in (4.8) for fixed $L$ with varying $D$ and fixed $D$ with varying $L$. 

### 4.2. Data

We used the $\mathtt{sc\_mixology}$ (Tian *and others,* 2019) scRNA-seq data set, which provides an experimentally derived “gold standard” true cell type identity (label) for each cell (https://github.com/LuyiTian/sc_mixology/). 

The UMI counts and cellular identities were obtained for $n$ = 902 cells comprised of three cell lines (H1975, H2228, and HCC827). The cell lines are used as the true cell type labels. Raw counts were $\log2$-normalized with a pseudocount of 1, and per-gene variance was calculated using $\mathtt{scran}$ (Lun *and others,* 2016). For comparison of distances, five dissimilarities (Euclidean, Maximum Manhattan, Canberra, and Binary) were calculated using $\log2$-normalized counts and the top 1000 most variant genes. For comparison of induced labels, dendrograms were induced directly from Euclidean distances using four hierarchical clustering methods (Ward’s method, single linkage method, complete linkage method, and unweighted pair group method with arithmetic mean). Cluster labels were induced by cutting each dendrogram at the true value of $k=3$. 

### 4.3. Fixed $L$ varying $D$

If a user were generating an analysis pipeline, prior to deployment, it may be insightful to compare the performance of several dissimilarity measures on a previously validated label–data set pair (Baker *and others,* 2021). In this case, fixing $L$ will imply that $\alpha_{m}=\alpha_{m-1}$, then from (4.8), we know that $s_{m}<s_{m-1}$ for two dissimilarity matrices $D_{m}$ and $D_{m-1}$. That is, the number of within-cluster distances greater than between-cluster distances will have strictly decreased. To illustrate this capacity, we used $H_{+}$ to compare the fitness of five dissimilarity methods induced from the same data and using the same “gold standard” true cell identities. These values may be found in Table S2 of the Supplementary material available at *Biostatistics* online. Further valuation of dissimilarities in this setting is outside the scope of this work, and we refer the reader to (Baker *and others,* 2021) for an exploration of this topic. 

### 4.4. Fixed $D$ varying $L$

**Fig. 5:**
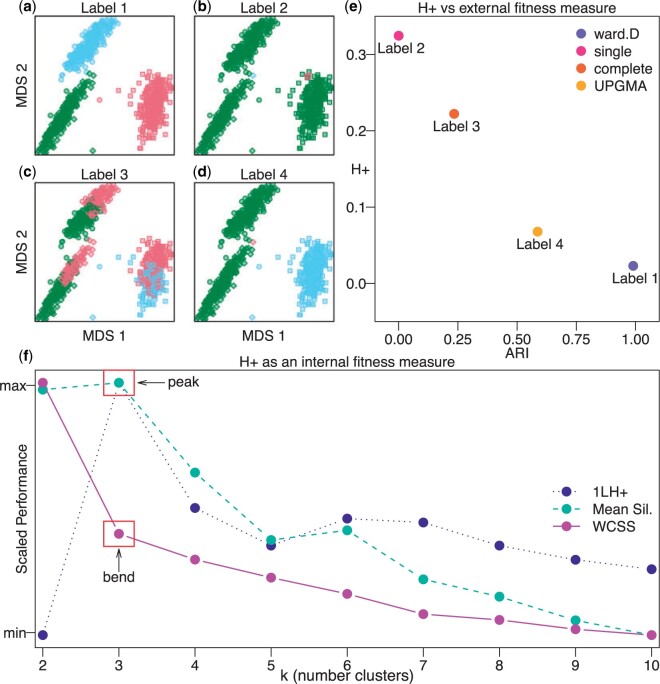
The $H_{+}$ metric is an internal validity measure for assessing the performance of induced cluster labels. Multidimensional scaling (MDS) plots with shapes representing true cell type labels from the $\mathtt{sc\_mixology}$ scRNA-seq data set and colors representing induced (or predicted) cluster labels from four hierarchical clustering methods implemented in the hclust() function in the base R stats package including (a) Ward’s method, (b) single linkage method, (c) complete linkage method, and (d) unweighted pair group method with arithmetic mean (UPGMA). (e) Scatter plot of $H_{+}$ (an internal validity metric) compared to Adjusted Rand Index (ARI) (an external validity metric) demonstrating shared information between the two metrics, which $H_{+}$ (calculated with the HPE algorithm 1 using $p=101$) recovers without the need of an externally labeled set of observations. (f) A performance plot with three internal validity metrics ($y$-axis scaled between 0 and 1): (i) $1-H_{+}$ (for ease of comparison) calculated from labels induced using with $k=2,\dots,10$ ($x$-axis), (ii) mean silhouette score, and (iii) within-clusters sums of square (WCSS). The “peak” of the $1-H_{+}$ metric at the correct $k=3$ indicates that $H_{+}$ accurately identifies the most accurate label in a comparable fashion to established internal fitness measure, namely a “peak” at the mean silhouette score and a “bend” in the WCSS curve.

Similarly, $D$ can be fixed (e.g., Euclidean distance) with the goal to compare the fitness of one generated label set $L_{m}$ (i.e., iteration $m$ of a clustering algorithm) to a previous label $L_{m-1}$. In this scenario, Equation (4.8) does not imply an explicit relation for $\alpha_{m},\alpha_{m-1}$; however, the discordance has still decreased. To demonstrate the use of $H_{+}$ as a cluster fitness metric, we induce labels using four hierarchical clustering methods (Ward’s method, single linkage method, complete linkage method, unweighted pair group method with arithmetic mean) (Figures 5(a–d)), and compare against well-known both external and internal validity metrics (Figure 5(e) and (f)). 

First, we compare $H_{+}$ as an internal validity metric to an external validity metric, namely the Adjusted Rand Index (ARI), which assesses the performance of the induced cluster labels using a gold-standard set of cell type labels in the $\mathtt{sc\_mixology}$ (Tian *and others,* 2019) scRNA-seq data set. Here, the induced labels with better (higher) ARI also yield better (less) $H_{+}$ discordance (Figure 5(e)). In this sense, $H_{+}$ (an internal validity measure without the dependency of a gold-standard set of labels) captures similar information as ARI (an external validity measure that depends on the use of a gold-standard set of labels). 

Next, we compare $H_{+}$ as an internal validity measure to other internal validity measures. Specifically, we induce labels using partition around medoids ($k$-medoids clustering) for values of $k=2,\dots,10$. For each label and $k$, the mean Silhouette score (Rousseeuw, 1987) and $H_{+}$ were calculated. We found that $H_{+}$ accurately identifies the correct $k=3$ for induced labels when compared to an internal validity metric (i.e., how well the data are explained by a single set of labels) using either the within-cluster sum of square “bend" (or “elbow") criterion or the mean Silhouette score (Figure 5(f)). 

## 5. Discussion

Quantifying how well a generated clustering fits the observed data is an essential problem in the statistical and computational sciences. Most methods for measuring cluster fitness are explicitly valued on the dissimilarity induced from the data. While appealing in their simplicity and interpretation, these approaches are potentially more susceptible to numerical bias between observations or types of dissimilarity measures. Discordance metrics, such as $\tau$ and $G_{+}$ circumvent this issue by assessing label-dissimilarity fitness implicitly on the dissimilarity values. In this work, we show $G_{+}$ is an estimator for the probability that a within-cluster dissimilarity is strictly greater than a between-cluster dissimilarity, $P(d_{ij}>d_{uv})$. However, we also show that $G_{+}$ varies as a function of the proportion of total distances that are within-cluster distances ($\alpha$) and thereby also the group balance ($b$) and number of groups $k$, which an undesirable property of the discordance metric. 

Here, we present $H_{+}$, a modification of $G_{+}$ that retains the scale-agnostic discordance quantification while addressing problems with $G_{+}$. Explicitly, $H_{+}$ is an unbiased estimator for $P(d_{ij}>d_{uv})$. This benefit is most easily seen in the manner that $H_{+}$ will be unaffected by the value of $\alpha$ (the portion of distance pairs that are within the same cluster), a formulation that permits the user to assess fitness for an arbitrary value of $k$. We discuss the theoretical properties of this estimator, provide two simple algorithms for implementation, and ascertain a strict numerical bound for their accuracy as a function of a simple user-defined parameter. We also introduce an estimator of $H_{+}$ based on bootstrap resampling from the original observations that does not require the full dissimilarity and adjacency matrices to be calculated. 

As $H_{+}$ can be used to assess the fitness of multiple dissimilarities for a fixed label, or to compare multiple labels given a fixed dissimilarity, we envision that $H_{+}$ can be employed in both development and analysis settings. If the true observation identities (labels) are known for a data set, $H_{+}$ could be utilized in the development stages of analytical software and pipelines to ascertain the most advantageous dissimilarity measure for that specific problem. In the alternate setting, we envision that $H_{+}$ can be used to quantify performance in clustering/classification scenarios. If the true labels are unknown, $H_{+}$ could be used to identify the clustering algorithm which produces the tightest clusters for a fixed dissimilarity measure. As a possible future direction, one could imagine directly minimizing discordance as the objective criteria within a clustering algorithm for optimizing iterative labels. 

Due to its generalizability to the number of clusters $k$ or the portion of within to within-cluster dissimilarity pairs $\alpha$, $H_{+}$ may be susceptible to degenerate cluster labels. For example, in the hierarchical clustering portion of Figure 5, Label 4 is less discordant than Label 3 in terms of both $H_{+}$ and ARI. Label 4 has simply merged two true clusters, and placed a single point in a third identity. While Label 4 is more accurate than Label 3, it achieves this by exploiting an opportunity to increase the proportion of same-cluster pairs, that is, maximizing $\alpha$. One could also imagine a scenario where an algorithm simply makes $k$ very large to minimize $\alpha$. In both scenarios, the labels generated are unlikely to be particularly informative for the user. We posit that some form of penalization for $H_{+}$ may help to alleviate these degenerate cases. For example, dividing $H_{+}$ by $\mathtt{max}\{\alpha,1-\alpha\}$ is a penalty for degeneracy in the case of putting many observations in the same label. Conversely, a division by $\mathtt{min}\{\alpha,1-\alpha\}$ is a potential penalty for the other degeneracy of making many very small clusters. 

We also imagine that discordance measures can be synthesized with probabilistic dissimilarity frameworks such as locality-sensitive hashing (LSH) and coresets (Datar *and others,* 2004; Har-Peled and Mazumdar, 2004). For example, it could be useful if theoretical (probabilistic) guarantees of observation proximity from LSH algorithms could be extended to similar guarantees for the discordance of observations embedded in the hash space. It may also prove fruitful to explore discordance outside the scope of the clustering/classification problem, such as pseudotime (1-dimensional ordering) or “soft” (weighting membership estimation) clustering problems. 

In practice, $H_{+}$ could provide an additional means to consider the termination of a clustering algorithm in a distance-agnostic manner. For example, the $k$-means algorithm (Hartigan and Wong, 1979) and its variants seek to minimize a form of the total within-cluster dispersion (dissimilarity). These algorithms with similar objective functions are subject to changes in behavior as the distance function changes. The extent to which minimizing discordance such as $H_{+}$ provides benefits regarding sensitivity to noise and magnitude of the distances is intriguing and outside the scope of this work. 

## Supplementary Material

kxac035_Supplementarey_Data

## Code and software availability

All analyses and simulations were conducted in the $\mathtt{R}$ programming language. Code for reproduction of all plots in this article is available at https://github.com/stephaniehicks/fasthpluspaper. Both HPE and HPB have been implemented in the $\mathtt{fasthplus}$ package in $\mathtt{R}$ available on CRAN at https://CRAN.R-project.org/package=fasthplus and for developmental versions on GitHub at https://github.com/ntdyjack/fasthplus.
