# Supplementary material for: A scalable and unbiased discordance metric with H+
Source: Biostatistics. 2022 Sep 5;25(1):188–202. doi: 10.1093/biostatistics/kxac035 (PMC10724244; doi:10.1093/biostatistics/kxac035)
Supplement: kxac035_Supplementarey_Data [file kxac035_supplementarey_data.pdf]

Supplementary Materials

A scalable and unbiased discordance metric with H_+

Nathan Dyjack, Daniel N. Baker, Vladimir Braverman, Ben Langmead, Stephanie C. Hicks*

*Correspondence to shicks19@jhu.edu

Contents

1. **Supplemental Notes.**
2. **Supplemental Figures S1-S2.**
3. **Supplemental Table S1-S2.**

Supplemental Notes

Note 1

Assume we have a set of n unique observations. For a given dissimilarity matrix D (e.g. Euclidean distance):

$$D = \begin{bmatrix} d_{11} & d_{12} & \cdots & d_{1n} \\ & d_{22} & \cdots & d_{2n} \\ & & \ddots & \vdots \\ & & & d_{nn} \end{bmatrix}$$

and fixed set of predicted cluster labels L, we can generate an adjacency matrix that tells us whether each observation has the same label (i.e., falls in the same cluster)

$$A = \begin{bmatrix} a_{11} & a_{12} & \cdots & a_{1n} \\ & a_{22} & \cdots & a_{2n} \\ & & \ddots & \vdots \\ & & & a_{nn} \end{bmatrix} \quad a_{ij} = \begin{matrix} 1 & \text{if } l_i = l_j \\ 0 & \text{otherwise} \end{matrix}$$

Using d_{ij} and a_{ij} for each i, j pairs of observations, we can then rewrite s in Equation 1 in terms of A, D. Let $D_W = \{d_{ij} : a_{ij} = 1; i = 2, \ldots, n, j < i\}$, that is, looping over the upper-triangular elements of A, D such that $a_{ij} = 1$ to select pairs which correspond to observations that are within the same cluster. We similarly define $D_B = \{d_{uv} : a_{uv} = 0; u = 2, \ldots, n, v < u\}$, the dissimilarity pairs corresponding to observations with different cluster labels.

$$\begin{aligned} s &= \sum_{d_{ij} \in D_W} \sum_{d_{uv} \in D_B} 1_{[d_{ij} > d_{uv}]} \\ &= \sum_{i=2}^{n} \sum_{j<i} 1_{[a_{ij}=1]} \sum_{u=2}^{n} \sum_{v<u} 1_{[a_{uv}=0]} 1_{[d_{ij} > d_{uv}]} \end{aligned}$$

The total number of distances in each of these sets is $|D_W|$ and $|D_B|$, respectively. As each upper triangular entry of A is binary (every distance is either between or within-cluster), we know that $|D_W| + |D_B| = N_d$. We can define α where $\alpha \in (0, 1)$ as the portion of total distances N_d that are within-cluster distances (or $d_{ij} \in D_W$). In this way, we can define $|D_W| = \alpha N_d$, and similarly, $|D_B| = (1 - \alpha) N_d$.

Conditional on N_d and α, the expected value of s is

$$\begin{aligned} E[s] &= E\left[\sum_{i=2}^{n} \sum_{j<i} 1_{[a_{ij}=1]} \sum_{u=2}^{n} \sum_{v<u} 1_{[a_{uv}=0]} 1_{[d_{ij} > d_{uv}]}\right] \\ &= E\left[\sum_{i=2}^{n} \sum_{j<i} 1_{[a_{ij}=1]}\right] E\left[\sum_{u=2}^{n} \sum_{v<u} 1_{[a_{uv}=0]}\right] E\left[1_{[d_{ij} > d_{uv}]}\right] \\ &= N_d P(a_{ij} = 1) N_d P(a_{uv} = 0) P(d_{ij} > d_{uv}) \\ &= \alpha(1 - \alpha) N_d^2 P(d_{ij} > d_{uv}) \end{aligned}$$

where $P(d_{ij} > d_{uv})$ is the probability a within-cluster distance $d_{ij} \in D_w$ is greater than a between-cluster distance $d_{uv} \in D_B$.

Then, the expectation of G_+ is:

$$\begin{aligned} E[G_+] &= \frac{E[s]}{N_d(N_d - 1)/2} \\ &= \frac{\alpha(1 - \alpha) N_d^2 P(d_{ij} > d_{uv})}{N_d(N_d - 1)/2} \\ &= \frac{N_d}{N_d - 1} 2\alpha(1 - \alpha) P(d_{ij} > d_{uv}) \end{aligned}$$

Note 2

We can also consider convergence in terms of the sum s by considering $q(D_W)$ and $q(D_B)$ as sampling without replacement from D_W and D_B. We denote s_e the estimated form of the sum s Equation 2, that is

$$s_e = \sum_{d_{ij} \in q(D_W)} \sum_{d_{uv} \in q(D_B)} 1(d_{ij} > d_{uv}) \tag{1}$$

While $|q(D_W)| \leq |D_W|$ and $|q(D_B)| \leq |D_B|$, we have that $s_e + s_n = s$ where s_n represents portions of the summand s that have not yet been counted in s_e. This allows us to consider the convergence of an estimated H_e to the true H_e in terms of the decomposition $s_e = s - s_n$

$$H_+ - H_e = \frac{s}{|D_W||D_B|} - \frac{s_e}{|q(D_W)||q(D_B|)} = \frac{s}{|D_W||D_B|} - \frac{s}{|q(D_W)||q(D_B|)} + \frac{s_n}{|q(D_W)||q(D_B)|} \tag{2}$$

The denominators of the second and third term approach $|D_W||D_B|$ as the number of distances sampled increases. The second term seems to approach H+ at $1/|q(D_W)||q(D_B)|$. The third term approaches zero as $|q(D_W)||q(D_B)| \to |D_W||D_B|$ and s_n decreases with each iteration in a factor bounded by $|q(D_W)||q(D_B)|$. This argument provides an intuitive argument that the convergence is achieved simply by increasing $|q(D_W)|$ and $|q(D_B)|$.

Supplemental Figures

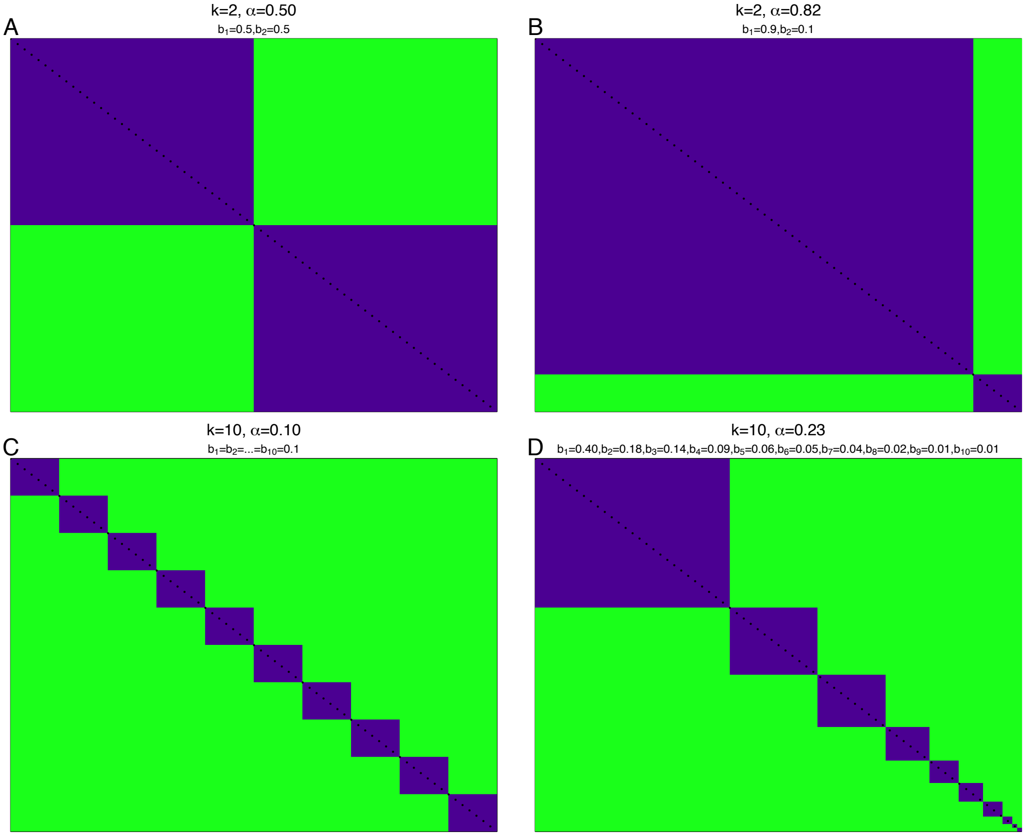

Supplementary Figure S1. An illustration of the relationship between group (or class) balance and α. Recall that α is the portion of total distances N_d that are within-cluster distances, or $|D_W| = \alpha N_d$, which can be seen most easily by using a heatmap of an adjacency matrix for **(A-B)** two or **(C-D)** ten groups for **(A,C)** balanced or **(B,D)** imbalanced groups. Unique pairs of observations are given on the upper-triangular potion of each adjacency matrix. Within-cluster pairs are given in blue, and between-cluster pairs are given in green. In other words, α is given by the blue proportion of each adjacency matrix. The group balance (and corresponding α) is **(A)** $b_1, b_2 = 0.5$ (α = 0.50), **(B)** $b_1 = 0.9, b_2 = 0.1$ (α = 0.82), **(C)** $b_1, \ldots, b_{10} = 0.1$ (α = 0.10), and **(D)** $b_1, b_2, \ldots, b_{10} = 0.40, 0.18, 0.14, 0.09, 0.06, 0.05, 0.04, 0.02, 0.01, 0.01$ (α = 0.23).

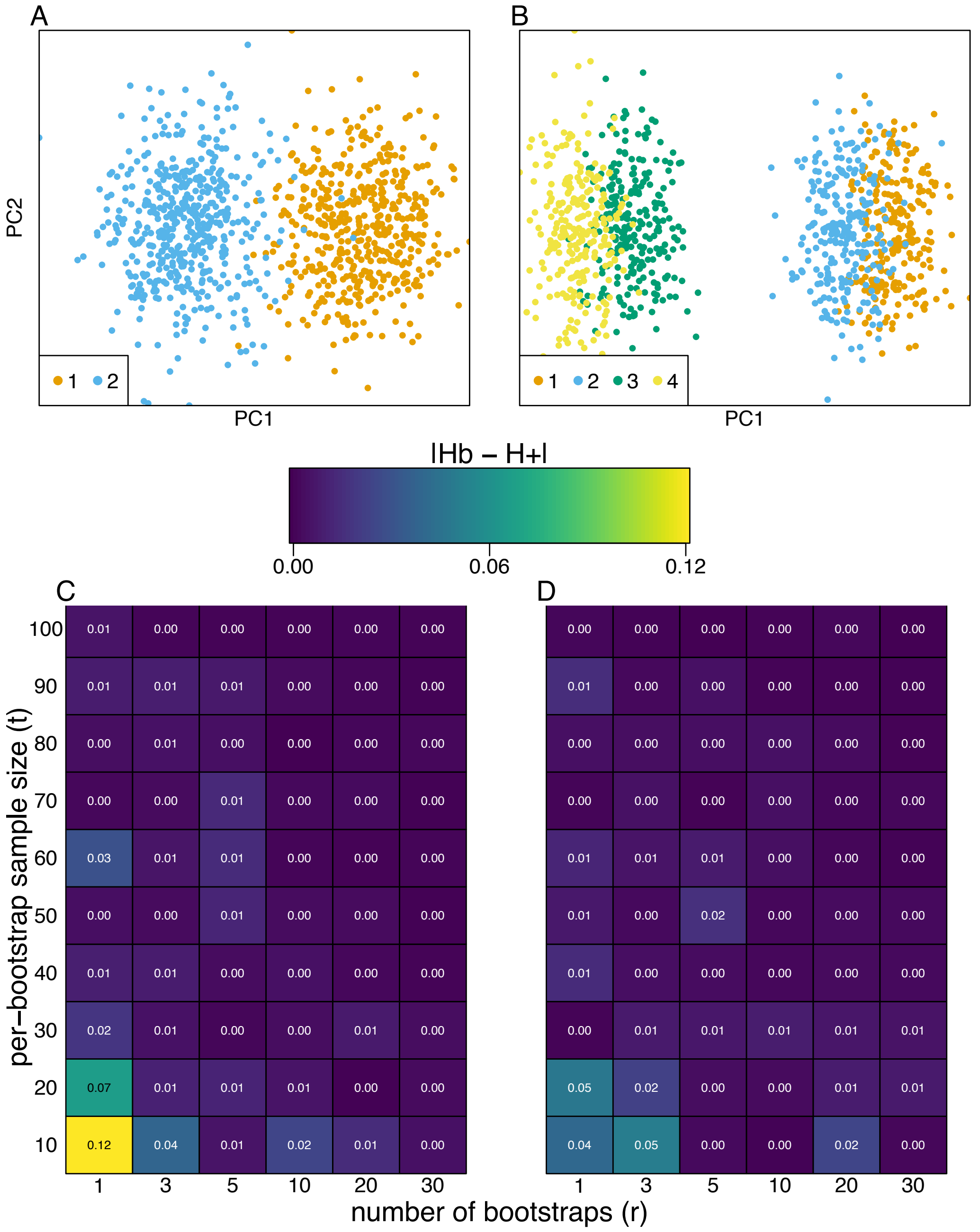

Supplementary Figure S2. **Accuracy of the bootstrap H_+ estimation procedure for two simulated datasets.** Two datasets were simulated using 1000 observations, 500 features with two $(N(-0.05, 0.25), N(0.05, 0.25))$ **(A)** and four $(N(-0.15, 0.25), N(-0.10, 0.25), N(0.10, 0.25), N(0.15, 0.25))$ **(B)** balanced classes. The difference absolute difference between H_+ (estimated using HPE with $p = 10001$) and the bootstrap estimate H_b for r replications (bootstraps) using s samples per bootstrap. For these simulations, sampling as little as 1% ($t = 10$) of the observations over $r = 30$ bootstraps provides an accurate estimate for H_+.

Supplemental Tables

Supplementary Table S1. Performance evaluation for elapsed time as reported in Figure 3. We report the elapsed time (seconds) for the individual components including calculating the dissimilarity matrix (dis), the adjacency matrix (adj), s (sum), HPE estimate using the `hpe()` function in the `fasthplus` R package, and HPB estimate using the `hpb()` function in the `fasthplus` R package for increasing sizes of datasets with n = 100, 500, 1,000, and 3,000 observations and 500 features. All observations were simulated from $N_{500}(0, 1)$ and then split evenly in two groups. The `hpb` procedure used $r = 0.05 \times n$ with $t = 30$, and the `hpe` procedure used $p = 1001$ with the grid search algorithm.

obs	100	500	1000	3000
dis	0.01	0.22	3.00	36.71
adj	0.00	0.01	0.08	0.44
sum	0.08	59.68	NA	NA
hpe	0.12	0.15	0.28	1.86
hpb	0.01	0.05	0.20	5.74

Supplementary Table S2. Performance evaluation of H_+ using known observation labels and several dissimilarity methods. We report estimated H_+ for fixed (experimentally validated *a priori*) labels of 902 scRNA-seq observations. For each of the 5 dissimilarity methods H_+ was estimated using the validated labels, and the `hpe` procedure with $p = 10001$.

Dissimilarity	H+
Euclidean	0.022
Maximum	0.124
Manhattan	0.021
Canberra	0.047
Binary	0.078
